# Sex Differences in Anxiety and Depression Conditions among Cancer Patients: A Systematic Review and Meta-Analysis

**DOI:** 10.3390/cancers16111969

**Published:** 2024-05-22

**Authors:** Elsa Vitale, Kurvatteppa Halemani, Asha Shetty, Yun-Chen Chang, Wen-Yu Hu, Raffaella Massafra, Annamaria Moretti

**Affiliations:** 1 Scientific Directorate, IRCCS Istituto Tumori “Giovanni Paolo II”, 70124 Bari, Italy; 2 College of Nursing, All India Institute of Medical Sciences (AIIMS), Raebareli 229405, India; halemani@aiimsrbl.edu.in; 3 College of Nursing, All India Institute of Medical Sciences (AIIMS), Bhubaneswar 751019, India; con_asha@aiimsbhubaneswar.edu.in; 4 School of Nursing and Graduate Institute of Nursing, China Medical University, Taichung 406040, Taiwan; lisacow@mail.cmu.edu.tw; 5 Nursing Department, China Medical University Hospital, Taichung 404328, Taiwan; 6 School of Nursing, College of Medicine, National Taiwan University, Taipei 106319, Taiwan; weyuhu@ntu.edu.tw; 7 Department of Nursing, National Taiwan University Hospital, Taipei 100225, Taiwan; 8 Laboratorio di Bioinformatica e Biostatistica, IRCCS Istituto Tumori “Giovanni Paolo II”, 70124 Bari, Italy; r.massafra@oncologico.bari.it; 9 Italian Group for Health and Gender, 70124 Bari, Italy; annamaria.moretti@uniba.it

**Keywords:** anxiety, cancer, depression, sex differences

## Abstract

**Simple Summary:**

The present systematic review and meta-analysis aimed to assess how anxiety and depression conditions among cancer patients vary according to sex. This systematic review and meta-analysis was registered in PROSPERO with id no. CRD42024512553. The search strategy involved combining keywords using Boolean operators, including “Anxiety”, “Cancer”, and “Depression”, across several databases: British Nursing Database, CINAHL, Embase, Nursing & Allied Health Database, PubMed, Scopus, and Web of Science. The outcomes were evaluated using the Hospital Anxiety and Depression Scale (HADS). Data were collected from five studies, enrolling a total of 6317 cancer patients, of whom 2961 were females and 3356 males. Generally, females reported significant higher levels of depression scores than males and, conversely, males reported significantly greater levels of anxiety than females.

**Abstract:**

(1) Background: Evidence suggested inconsistent results in anxiety and depression scores among female and male cancer patients. The present systematic review and meta-analysis aimed to assess how anxiety and depression conditions among cancer patients vary according to sex. (2) Methods: This systematic review and meta-analysis was conducted according to the Preferred Reporting Items for Systematic Reviews and Meta-analysis (PRISMA). The protocol was registered in PROSPERO with id no. CRD42024512553. The search strategy involved combining keywords using Boolean operators, including “Anxiety”, “Cancer”, and “Depression”, across several databases: Embase, PubMed, Scopus, and Web of Science. The outcomes were evaluated using the Hospital Anxiety and Depression Scale (HADS). (3) Results: Data were collected from five studies, enrolling a total of 6317 cancer patients, of whom 2961 were females and 3356 males. For each study, HADS-A and HADS-D scores were considered, also differentiating HADS scores according to cancer typology, and then three different meta-analyses were performed. Generally, females reported significantly higher levels of depression scores than males and, conversely, males reported significantly greater levels of anxiety than females. (4) Conclusions: Previous studies suggested higher rates of depression and anxiety conditions in females than in males, but the present data highlighted controversial findings, since males reported significantly higher levels of anxiety than females. In this scenario, the theoretical approach justified females being more open than males to expressing anxiety or depression conditions. It would be necessary for healthcare professionals to improve effective measures purposed at assessing and mitigating depressive symptoms in cases of advanced cancer, thereby improving their mental health, given the high rates of depression in advanced cancer patients, due to the difficulty level of performing their daily living activities, which deteriorate further over time.

## 1. Introduction

Cancer is a leading cause of death worldwide, accounting for nearly 10 million deaths in 2020, or nearly one in six deaths. The most prevalent types of cancer are those of the breast, lung, colon and rectum, and prostate [1]. The reported increase in incidence may be partially explained by an increase in risk behavior in terms of excessive tobacco use, high body mass index, alcohol consumption, low fruit and vegetable intake, and lack of physical activity [1].

Despite oncology improvements, cancer is still commonly overlapped with the death concept, pain, and affliction [2]. Cancer is not only a unique incident with a persistent condition distinguished by constant ambiguity and the delayed consequences of the disease, but it also has care and concomitant psychological concerns [3]. Various physical and psychological troubles have been considered as direct consequences of neoplasms, and the treatment dimensions have been often considered as interference with the patients’ daily activities and skills to preserve family and social relationships [4]. Past researchers have just explained a positive association between cancer diseases and psychiatric ones [5,6], since cancer diagnosis and treatment have generally impacted on distress and negatively interfered in daily activities [7], with increasing rates in new diagnoses of clinical reports of anxiety (10 to 58%) and depression (14 to 30%) [8,9]. However, data from the current literature seem to be inconsistent, since there are some studies which report decreases in anxiety and depression symptoms [10] and others which report worsened depression conditions associated with a cancer diagnosis [11]. However, further researchers present reasonable evidence that cancer patients with a psychiatric morbidity, such as depression or anxiety symptoms, appear to record poor survival and a lower quality of life [12,13]. Additionally, in a healthy general population, particularly among sexual minorities, it has been registered that there is nearly a 1.5 times higher risk of anxiety and depression than in the heterosexual population [14,15], with worse anxiety and depression levels being reported. In a systematic review involving cancer patients, females report higher levels of depression than males; however, other studies suggest the contrary [12]. In cases of advanced cancer, women and younger patients record higher depressive symptoms than men and older individuals [16,17]. Additionally, social characteristics could also play a determinant role in patients’ emotional condition [18,19]. In fact, females, younger patients, and unmarried individuals with recurring cancer show more depressive symptoms than males, older patients, married patients, and those discovering cancer for the first time [16,17].

By considering all the abovementioned literature, the present systematic review and meta-analysis aims to assess how anxiety and depression conditions vary among cancer patients according to sex.

## 2. Materials and Methods

### 2.1. Search Strategy

The present systematic review and meta-analysis was conducted according to the Equator checklist for reviews, like the Preferred Reporting Items for Systematic Reviews and Meta-analysis (PRISMA) [20]. The protocol was registered in PROSPERO with id no. CRD42024512553.

Keywords and MeSH expressions were combined with the Boolean operators, such as “Anxiety”, “Cancer”, and “Depression” (Supplementary Materials File S1), and the “Population-Intervention-Outcome” (PIO) arrangement was built (Table 1). The Embase, PubMed, Scopus, and Web of Science databases were consulted for this review.

The review included all the interventional (randomized clinical trials, quasi-experimental) studies recording anxiety and depression conditions with the Hospital Anxiety and Depression Scale (HADS) [21,22,23] among cancer patients. Specifically, the HADS covers a total of 14 items. Of these, 7 items explore anxiety (HADS-A) and 7 ones quantify depression (HADS-D). Each subdimension ranges from 0 to 21 and higher scores indicate a greater distress level. Evidence suggests a high reliability and validity of the scale for both of the two subdimensions. Additionally, the present review only included manuscripts published in the English language available in their full-text versions.

### 2.2. Peer Review and Data Extraction

Initially, records were identified through a systematic database search, uploaded to reference management software, and duplicate studies were removed. Then, two independent reviewers (E.V. and H.K.) assessed the title and abstract of the identified studies, inclusion was assessed, and unsuitable reports were removed. After that, articles were uploaded, and the full-text version of each article was assessed more closely according to the fixed eligibility criteria. Disagreements about whether a study should be included or not were resolved by discussion and consensus. If the disagreement remained, arbitration from another reviewer was provided. Data collection was performed by considering the following: study characteristics (author, year of publication, aim, design, sample size, and setting), participants (age, cancer stage, and type of treatments performed), and the outcomes both in anxiety and depression were also appropriately assessed by the HADS [21].

### 2.3. Records Assessed

**Figure 1:**
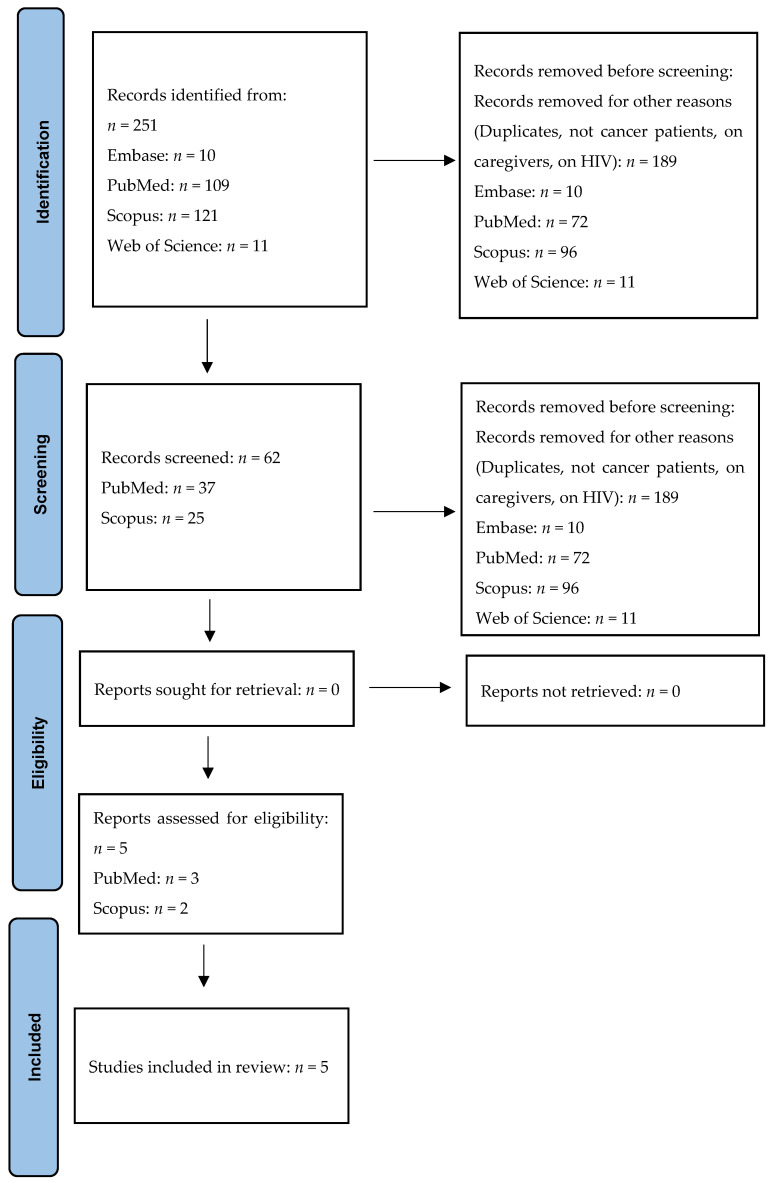
The PRISMA flow-chart.

A total of 251 records were searched at the first phase of the present literature review (Figure 1). Then, 189 articles were removed as their titles were not in accordance with the purpose of this review. After selecting the remaining articles, a total of 57 eligible records were further screened, since their abstracts did not meet all the inclusion criteria fixed in our protocol, as both anxiety and depression were not assessed through the HADS, the participants were cancer patients’ caregivers, patients did not suffer from a cancer disease, manuscripts were written in other languages different from the English one, or the full-text version was not available. Additionally, the same manuscripts present in all the databases considered were screened and taken into consideration only once. Thus, only 5 articles were considered as eligible for the present systematic review and meta-analysis (Figure 1).

### 2.4. Interventions and Outcomes

The present systematic review and meta-analysis embraced all interventional, randomized clinical trials, and quasi-experimental studies recording anxiety and depression conditions with the Hospital Anxiety and Depression Scale (HADS) [21], before and after a coaching intervention in any cancer treatment, such as surgery, chemotherapy, radiotherapy, or endocrine therapy. The HADS includes a total of 14 items, of which 7 items assess anxiety (HADS-A) and 7 items quantify depression (HADS-D) [22]. Each subdimension ranges from 0 to 21 and higher scores indicate a greater pathological condition level. Evidence suggests a high reliability and validity of the scale for both the two subdimensions investigated [23]. Although the literature suggested several anxiety and depression self-reported questionnaires, we chose the HADS self-reported questionnaire as our outcome tool as it reports both anxiety and depression in a unique questionnaire [23].

No pre-existing psychiatric conditions were highlighted: all studies included considered only health psychiatric patients, who, after a cancer diagnosis, might consequentially develop an anxious or depressive disorder.

Additionally, data were collected from patients who self-reported their sex identities as female/male.

### 2.5. Quality and Bias Assessments

The quality assessment of all the selected studies was assessed by considering the study design of each manuscript included in the present review, in accordance with the Evidence-Based Nursing (EBN) approach [24]. The EBN methodology included a total of seven levels of evidence, ranging from I to VII, suggesting the weakest quality of study design, specifically as follows:

- Level I: Evidence from systematic reviews or meta-analysis of randomized control trials;
- Level II: Evidence from well-designed randomized control trials;
- Level III: Evidence from well-designed control trials that are not randomized;
- Level IV: Evidence from case–control or cohort studies;
- Level V: Evidence from systematic reviews of descriptive or qualitative studies;
- Level VI: Evidence from a single descriptive or qualitative study;
- Level VII: Evidence from expert opinions.

Furthermore, due to the nature of primary studies selected, we employed the Downs and Black checklist [25] to evaluate the quality of the included studies. This instrument evaluates the quality of both randomized and non-randomized studies. The tool consists of 27 items with 3 related subheadings: external validity, internal validity, and power. Based on these assessments, the tool has the categories of “low” (score less than 14), “fair” (score between 15 and 19), “good” (score between 20 and 25), and “excellent” (score between 26 and 27) [25].

### 2.6. Main Outcome(s)

The Hospital Anxiety and Depression Scale (HADS) was used to assess differences in cancer patients between females and males, and then we considered the cancer typologies included in the literature.

### 2.7. Measures of Effect

Previous studies showed that the HADS contains a total of 14 items on a self-rating scale with two subdimensions, one assessing anxiety (HADS-A) and the other one assessing depression (HADS-D). Anxiety and depression cutoff values for pathological conditions ranged from 8 to 11 in oncologic settings and the data were presented as means and standard deviations [26].

### 2.8. Data Synthesis

Studies were assessed for quality as per protocol recommendations. The information retrieved from the final selected studies was expressed using both a narrative approach and tables. Descriptive statistics were presented as means, standard deviations, and number of cases (n) in a Forest plot. Statistical analysis was performed using the R environment (version 4.2). A random effects meta-analysis based on estimates and their standard errors was implemented. To assess the consistency across studies, the I^2^ statistic was adopted with 25%, 50%, and 75% suggesting low, moderate, and high heterogeneity degrees, respectively. However, I^2^ should be presented and interpreted cautiously in small meta-analyses [27]. For this reason, 95% confidence intervals (95% CI) were presented in addition to the point estimate. The χ^2^-based Q test was also applied to look for the heterogeneity of effects among studies. The Q statistic at a cut-off significance level of 0.10 suggested the presence of significant heterogeneity. The τ^2^ statistic was also presented to check the variance of the true effects.

## 3. Results

Data were collected from five studies [28,29,30,31,32], enrolling a total of 6317 cancer patients, of whom 2961 were females and 3356 males. For each study, HADS-A and HADS-D scores were considered, HADS scores were differentiated according to cancer typology, and then three different meta-analyses were performed.

Table 2 explained all study characteristics, specifically the following: author(s) and year of publication, study design, level of evidence, sample size according to sex, range of age, eligible criteria, instruments to assess both anxiety and depression, and findings revealed.

### 3.1. Study Characteristics

In the Skarstein et al. study [28], a total of 568 cancer patients were enrolled and simultaneously evaluated with the Research and Treatment of Cancer Quality of Life Questionnaire (EORTC QLQ C33) and the Hospital Anxiety and Depression Scale (HADS). Statistically significant negative associations were assessed between HADS-A, HADS-D, and HADS-T (total score). Males of all ages recorded worse HADS scores than females. In this regard, Nipp et al. [31] showed that males and younger patients receiving early palliative care assistance better perceived their quality of life and anxiety than those receiving usual oncology care. Conversely, females and older patients did not report any difference in their treatment impacts.

Evidence reported that nearly 47.2% and 57% of gastrointestinal cancer patients reported high levels of both anxiety and depression. However, there were no significant differences between sex, while patients who knew their cancer diagnosis reported very high anxiety and depression scores [29]. Moser et al. [30] considered that being female could inevitably be a risk factor for high perceived depression and anxiety levels. In this regard, they assessed a significant proportion of patients reporting high levels of anxiety, depression, and psychological distress, among females and both among patients and partners, too, by assigning to the female sex a negative trend in psychological conditions among cancer patients. Also, Oertelt-Prigione et al. [32] reported negative associations in emotional functions and female survivors, since female survivors had more moderate physical and cognitive functioning than males who reported a significant loss in their social roles. Therefore, a significant and unexpected long-term impact on male patients was reported by highlighting the need to further investigate these sex-related dimensions.

### 3.2. Bias Risk Assessment

In addition to this, we employed the Downs and Black checklist [25] to assess the quality of randomized trials and non-controlled studies. Two reviewers (E.V. and K.H.) independently assessed the quality of studies under the following headings: reporting, external validity, and internal validity (bias and confounding) against the rater checklist (Downs and Black checklist to 0.1.2). After the quality assessment, both reviewers mutually checked individual studies’ scores, and the third author (A.S.) sorted out any disagreements between the first two reviewers (E.V. and K.H.). This Downs and Black checklist included a total of 28 items under three headings, reporting, internal validity, and extremal validity, referring to the power of the study rating and whether or not the study was performed to the power calculation. The maximum score for each item was 1, as a power analysis assessed the five studies, and thus the highest possible score for the checklist was 28. The Downs and Black score varied according to the quality levels as follows: excellent (26–28); good (20–25); fair (15–19); and poor (≤14). To assess bias risk, the reviewers’ results were compared by an external reviewer, and discrepancies were resolved in a consensus meeting (Table 3).

### 3.3. Meta-Analysis Results for HADS-Anxiety (HADS-A)

**Figure 2:**
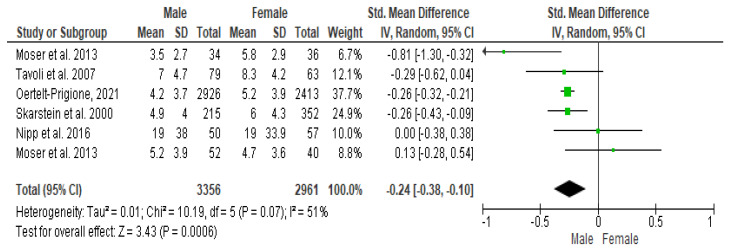
Forest plot of HADS-A between female and male cancer patients. The Cochrane Q-test explained the presence of a significant heterogeneity between the studies selected.

**Figure 3:**
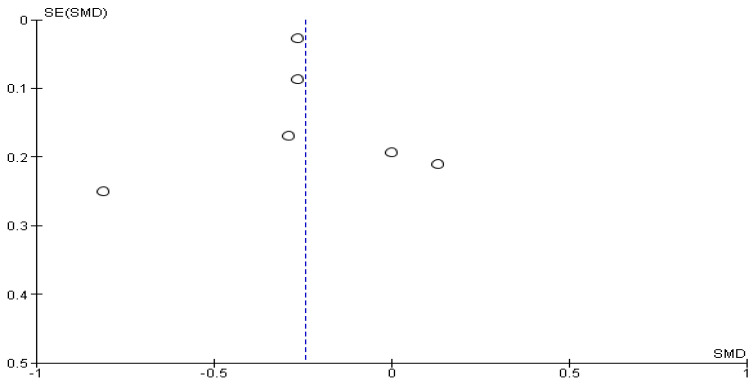
Publication bias of HADS-A between female and male cancer patients. The absence of publication bias and Egger’s regression intercept confirmed this result. A significant difference between the two sexes (Z = 3.43; *p* = 0.0006) confirmed that males recorded significantly higher values of anxiety than females.

The Cochrane Q-test revealed the presence of a significant heterogeneity between the studies (*P*_Q_ = 0.001; tau^2^ = 0.01; I^2^ = 51%) (Figure 2). Figure 3 suggests the absence of publication bias, and Egger’s regression intercept confirmed this result (β = −0.24; 95% CI = [−0.38–0.10]). Finally, the overall effect showed a significant difference between the two sexes (Z = 3.43; *p* = 0.0006), as males recorded significantly higher values of anxiety than females.

### 3.4. Meta-Analysis Results for HADS-Depression (HADS-D)

**Figure 4:**
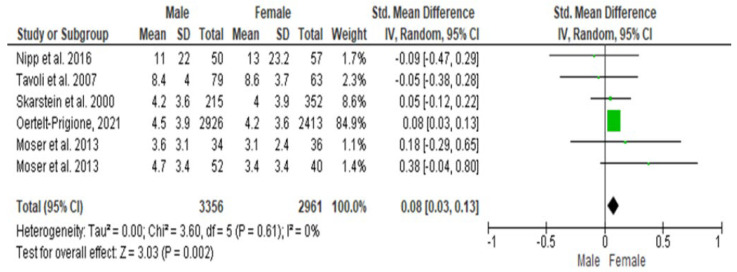
Forest plot of HADS-D between female and male cancer patients. The Cochrane Q-test revealed the presence of a significant heterogeneity between the selected studies.

**Figure 5:**
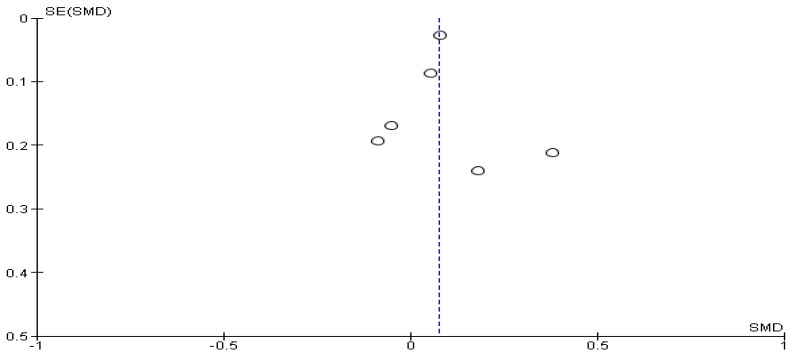
Publication bias of HADS-D between female and male cancer patients. Figure 5 suggested the absence of publication bias, and the regression intercept confirmed this result: females recorded significantly higher values of depression scores than males.

The Cochrane Q-test revealed the presence of a significant heterogeneity between the studies (*P*_Q_ = 0.61; tau^2^ < 0.001; I^2^ = 0%) (Figure 4). Figure 5 suggests the absence of publication bias, and the regression intercept confirmed this result (β = 0.08; 95% CI = [0.03–0.13]). Finally, the overall effect showed a significant difference between the two sexes (Z = 3.03; *p* = 0.002), as females recorded significantly higher values of depression scores than males.

### 3.5. Meta-Analysis Results for HADS-Anxiety (HADS-A) among Patients Diagnosed with Gastrointestinal, Blood, and Thyroid Cancers

**Figure 6:**
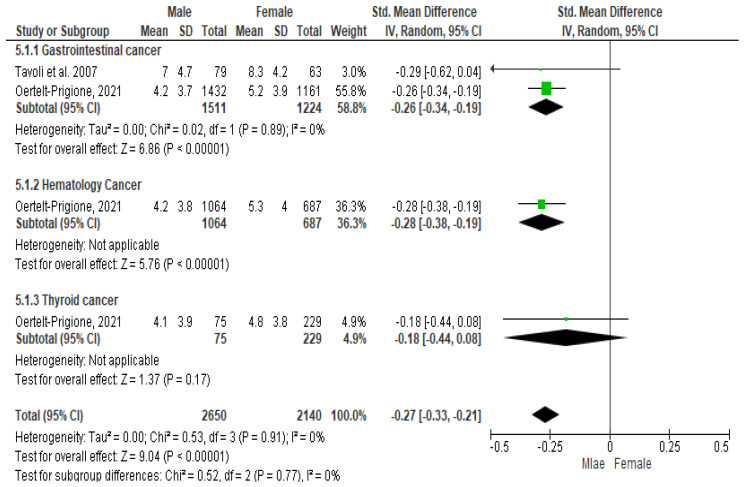
Anxiety symptoms among patients diagnosed with gastrointestinal, blood, and thyroid cancers.

As regards gastrointestinal cancer, the Cochrane Q-test revealed the presence of a significant heterogeneity between the selected studies (*P*_Q_ = 0.02; tau^2^ < 0001; I^2^ = 0%) (Figure 6). The Funnel plot suggested the absence of publication bias (Figure 6), and Egger’s regression intercept confirmed this result (β = −0.26; 95% CI = [−0.34–0.191]). Finally, the overall effect showed a significant difference between the two sexes (Z = 6.86; *p* < 0.00001), as males recorded significantly higher values of depression than females.

As regards hematologic cancers, the presence of a significant heterogeneity between the studies was not assessed, since there was only one article for this typology (Figure 4). The Funnel plot suggested the absence of publication bias (Figure 6) and the Egger’s regression intercept confirmed this result (β = −0.28; 95% CI = [−0.38–0.19]), too. Finally, the overall effect showed a significant difference between the two sexes (Z = 6.86; *p* < 0.00001), as males recorded significantly higher values of anxiety than females.

As regards thyroid cancer, the presence of a significant heterogeneity between the studies was not assessed, since there was only one article for this typology (Figure 4). The Funnel plot suggested the absence of publication bias (Figure 6) and the Egger’s regression intercept confirmed this result (β = −0.18; 95% CI = [−0.44–0.08]). Finally, the overall effect showed a significant difference between the two sexes (Z = 1.37; *p* = 0.17), as males recorded significantly higher values of anxiety than females.

### 3.6. Meta-Analysis Results for HADS-Depression (HADS-D) among Patients Diagnosed with Gastrointestinal, Blood, and Thyroid Cancers

**Figure 7:**
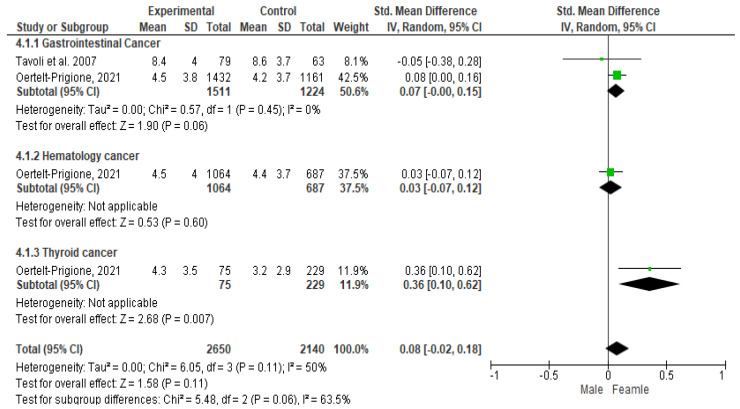
Depression symptoms among patients diagnosed with gastrointestinal, blood, and thyroid cancers.

As regards gastrointestinal cancer, the Cochrane Q-test revealed the presence of a significant heterogeneity between the studies (*P*_Q_ = 0.57; tau^2^ < 0001; I^2^ = 0%) (Figure 7). The Funnel plot suggested the absence of publication bias (Figure 5) and the Egger’s regression intercept supported this result (β = 0.07; 95% CI = [−0.00–0.15]). Finally, the overall effect showed a significant difference between the two sexes (Z = 1.90; *p* = 0.06), as females recorded significantly higher values of anxiety than males.

In hematologic cancers, the presence of a significant heterogeneity between the studies was not assessed, since there was only one article for this typology (Figure 7). The Funnel plot suggested the absence of publication bias (Figure 5) and the Egger’s regression intercept endorsed this finding (β = 0.03; 95% CI = [−0.07–0.12]). Finally, the overall effect did not show any significant difference between the two sexes (Z = 0.53; *p* = 0.60), as males and females recorded overlapping values of depression outcomes.

As regards thyroid cancer, the presence of a significant heterogeneity between the studies was not assessed, since there was only one article for this typology (Figure 5). The Funnel plot suggested the absence of publication bias (Figure 5) and the Egger’s regression intercept supported this result (β = 0.36; 95% CI = [0.10–0.62]). Finally, the overall effect showed a significant difference between the two sexes (Z = 2.68; *p* = 0.007), as females recorded significantly higher values of depression than males.

## 4. Discussion

The present systematic review and meta-analysis aimed to assess how anxiety and depression conditions vary between female and male cancer patients. The literature has explained how anxiety and depression have been commonly mentioned in psychological distress, due to the diagnosis of cancer [33], and most cancer patients have different levels of psychological difficulty, which depends on the progression of cancer [34]. In this regard, the American Psychiatric Association declared that individuals respond differently to the diagnosis of neoplasms; while some show specific anxious or depressive symptoms, others simultaneously experience both of the psychiatric conditions. However, each individual might respond differently thanks to different innate predispositions to handle the disease condition encountered [35].

In our systematic review and meta-analysis, data were collected from five studies [28,29,30,31,32] enrolling a total of 6317 cancer patients, of whom 2961 were females and 3356 males. For each study, HADS-A and HADS-D scores were considered, HADS scores were differentiated according to cancer typology, and then three different meta-analyses were performed. Generally, females reported significantly higher levels of depression scores than males and, conversely, males reported significantly greater levels of anxiety than females. Our data partially agreed with the Götze et al. study [36], in which moderate-to-severe depression and anxiety levels were reported in 17% and 9% of cancer survivors, with significant differences between females and males, since there were higher depression and anxiety levels (*p* < 0.001). Even Mitchell et al. highlighted a frequency of 17.9% for anxiety and 11.6% for depression among long-term cancer survivors (2 years after “any” cancer diagnosis) [8]. Linden et al. [37] showed that younger females registered more clinical and sub-clinical conditions of anxiety and depression than other patients, who better managed their emotions after their cancer diagnosis. Additionally, van’t Spijker et al.’s findings [38] suggested that females recorded lower rates of emotional distress, especially among female cancer patients recording higher prevalence rates of anxiety and depression than males. These data were in agreement with the high rates of anxiety and depression among healthy females compared to males [39]. This sex difference appeared to be in accordance with the concept regarding females who adopt emotional-related coping strategies [40,41].

However, as regards the anxiety dimension, our meta-analyzed data suggested that males recorded higher levels in the anxiety dimension than females. These data were in disagreement with the Hasan et al. study [34] in which females suffering from gynecological, hematological, head and neck, and lung cancers recorded significant higher levels of anxiety.

Moreover, anxiety could be positively associated with mental disengagement, negation, and requiring emotional social helping. In this regard, anxiety also showed a comparable association with a positive explanation, attitudinal detachment, and requiring emotional social support [42]. By considering the depression condition, our data could be explained by Hasan et al.’s study (2020), since depressed males could be positively correlated with mental disengagement and requiring social and emotional supports. In females, depression was positively correlated with behavioral detachment as well as negation [42]. However, by considering cancer typology, our meta-analysis showed that males recorded significantly higher values in the anxiety condition than females, in gastrointestinal, hematologic, and thyroid cancers. On the other hand, this trend seemed to be dissimilar when considering the depression condition, as in both gastrointestinal and thyroid cancer typologies, females recorded significantly higher values in the depression condition than males. This difference seemed to be overlaid by considering the hematologic cancer typology, since the overall effect did not show any significant difference between the two sexes. In this regard, previous studies highlighted several elements impacting on the anxiety and depression prevalence among cancer patients. However, very limited evidence was available among gastric cancer patients [43]. In this regard, Nordin et al. [44] reported 17% of gastrointestinal patients suffering from anxiety and 21% from depression overall [45,46]. Among hematologic cancer patients, Kuba et al. [47] reported significant differences in anxious and depressive symptomatologies. However, they did not consider sex-related differences. On the other hand, patients suffering from thyroid cancer experienced high levels of distress and worry, despite having a good prognosis, and reported more anxiety and depression rates than the other cancer typologies [48]. Further epidemiological studies [49,50] highlighted a positive association between anxiety and sex and age. However, our data were inconsistent with the current literature, since males reported significantly higher levels in the anxiety condition, and, for example, Noto et al. [51] considered female sex as a predictor for anxiety in thyroid cancer survivors.

### Strengths and Limitations

The present study showed interesting findings in sex-related differences in anxiety and depression conditions. The literature data collected were dated from 2007 to 2021, highlighting the necessity of an additional study to bring this up to date in the next few years. We also tried to consider age, and all the participants included in our screened manuscripts were aged between 19 and 92 years.

However, another variable which will be considered in future studies is whether or not cancer patients were followed by a psychological/psychiatric service to better investigate how cancer patients are accomplished in their care pathways in cancer disease. Another limit could be that screening for anxiety or depression at the diagnosis communication stage could be very different from a chronic condition, too.

## 5. Conclusions

Since previous studies suggested higher rates in the depression and anxiety conditions among females than in males, the present data highlighted controversial findings, since males reported significantly higher levels of anxiety than females.

In this scenario, the theoretical approach [52] justified females being more open than males to expressing anxiety or depression conditions. In addition, males seemed to have more difficulties in expressing their emotions or in requiring assistance. In fact, they tended to experience more different symptoms associated with stress conditions, such as anger, obsession, and hostility. Hegemonic masculinity could be explained as a potential cause that could hinder males to require psychological support, in opposition to females who have always required psychological support [53]. In this scenario, the progression of depression and anxiety generally seemed to be more inclined to females and commonly underestimated in males [54]. In this scenario, it would be necessary for healthcare professionals to improve effective measures purposed at assessing and mitigating depressive symptoms in cases of advanced cancer, thereby improving their mental health, given the high rates of depression in advanced cancer patients [55,56,57], due to the difficulty level in performing their daily living activities [58], which deteriorate further over time [59].

## Figures and Tables

**Table 1:** The PIO tool for the present systematic review and meta-analysis.

Population	Cancer patients
Intervention	Anxiety and depression assessments
Outcome	Anxiety and depression between females and males

**Table 2:** Anxiety and depression assessed with the HADS in oncology, comparing females and males (*n* = 5).

Name of Author, Year and Country	Design Level of Evidence	Sample Size	Age (min.–max. Years)	Eligible Criteria		Instruments	Findings
				Inclusion	Exclusion		
Skarstein et al., 2000 Norway [28]	Comparative study III level	F: 215 M: 353 T: 568	15–92 (median: 55)	Any type of cancer	NR	HADS-A HADS-D	Older male patients reported less distress than younger patients. Anxiety and depression directly impact on QoL.
Tavoli et al., 2007 Iran [29]	Cross-sectional study III level	F: 63 M: 79 T: 142	39–69	GI	Patients with cognitive problems	HADS-A HADS-D	Patients who knew their diagnosis reported higher distress. Family background and kind of information improve the psychological outcome.
Moser et al., 2013 Switzerland [30]	Cohort study IV level	F: 53 M: 69 T: 122	F: 30–84 M: 26–89	Newly diagnosed cancer patient	NR	HADS-A HADS-D QOL SCL-K9	Males experienced greater psychology problems than females.
Nipp et al., 2016 USA [31]	RCT II level	F: 50 M: 57 T: 107	>65 <65	NSCLC metastatic patient English language knowledge	Patients addressed to palliative care	FACT-L HADS-A PHQ-9	Male and younger patients had better QoL and anxiety when they received early palliative care.
Oertelt-Prigione et al., 2021 The Netherlands [32]	Cohort study IV level	F: 2413 M: 2926	40–70	Colorectal cancer Blood basal cell/squamous cell Thyroid cancer Dutch language knowledge	Impaired cognitive functions	EORTC HADS-A HADS-D SCQ.	Male patients reported more severe symptoms than female patients.

Abbreviations: EORTC Core Quality of Life questionnaire; F: Female; FACT-L: Functional Assessment of Cancer Therapy–Lung; GI: Gastrointestinal; HADS: Hospital Anxiety and Depression Scale; PHQ-9: Patient Health Questionnaire; HADS-A: The Hospital Anxiety and Depression Scale of Anxiety; HADS-D: The Hospital Anxiety and Depression Scale of Depression; NR: Not Reported; NSCLC: non-small-cell lung cancer; QOL: Quality of Life; M: Male; RCT: Randomized Controlled Trial; SCL-K-9: Symptom-Checklist-K-9; SCQ: Self-administered Comorbidity Questionnaire; T: Total.

**Table 3:** Quality assessment of all the studies included in the present systematic review.

Author(s) Publication Year	Country	Reporting (Total Score: 10)	External Validity (Total Score: 3)	Internal Validity-Confounding (Selection Bias)	Total Quality Score
Skarstein et al., 2000 [27]	Norway	8	3	12	23
Tavoli et al., 2007 [28]	Iran	7	3	11	21
Moser et al., 2013 [29]	Switzerland	8	2	10	20
Nipp et al., 2016 [30]	USA	8	2	12	22
Oertelt-Prigione et al., 2021 [31]	The Netherlands	7	3	11	21

Scoring: ≥14: poor; 15–19: fair; 20–25: good; 26–27: excellent.

## Data Availability

Data are available upon reasonable request to the corresponding author. Figures and tables within the manuscript are original.

## Supplementary Materials

The following supporting information can be downloaded at https://www.mdpi.com/article/10.3390/cancers16111969/s1, File S1: Search strings carried out to perform this systematic and meta-analysis study.
