# Supplementary material for: Sex Differences in Anxiety and Depression Conditions among Cancer Patients: A Systematic Review and Meta-Analysis
Source: Cancers (Basel). 2024 May 22;16(11):1969. doi: 10.3390/cancers16111969 (PMC11171373; doi:10.3390/cancers16111969)
Supplement: Supplementary file 1 [file cancers-16-01969-s001.zip › cancers-2960324-supplementary.pdf]

Supplementary File S1. Search strings carried out to perform this systematic and meta-analysis study.

Database	Results
**Embase**<br>('depression'/exp OR 'depression') AND ('neoplasms'/exp OR 'neoplasms') AND ('patients'/exp OR 'patients') AND ('sex role'/exp OR 'sex role')	10
**PubMed**<br>Search: (((Depression) AND (Neoplasms)) AND (Patients)) AND (Gender Role)<br>("depressed"[All Fields] OR "depression"[MeSH Terms] OR "depression"[All Fields] OR "depressions"[All Fields] OR "depression s"[All Fields] OR "depressive disorder"[MeSH Terms] OR ("depressive"[All Fields] AND "disorder"[All Fields]) OR "depressive disorder"[All Fields] OR "depressivity"[All Fields] OR "depressive"[All Fields] OR "depressively"[All Fields] OR "depressiveness"[All Fields] OR "depressives"[All Fields]) AND ("neoplasm s"[All Fields] OR "neoplasms"[MeSH Terms] OR "neoplasms"[All Fields] OR "neoplasm"[All Fields]) AND ("patient s"[All Fields] OR "patients"[MeSH Terms] OR "patients"[All Fields] OR "patient"[All Fields] OR "patients s"[All Fields]) AND ("gender role"[MeSH Terms] OR ("gender"[All Fields] AND "role"[All Fields]) OR "gender role"[All Fields])<br>Translations<br>Depression: "depressed"[All Fields] OR "depression"[MeSH Terms] OR "depression"[All Fields] OR "depressions"[All Fields] OR "depression's"[All Fields] OR "depressive disorder"[MeSH Terms] OR ("depressive"[All Fields] AND "disorder"[All Fields]) OR "depressive disorder"[All Fields] OR "depressivity"[All Fields] OR "depressive"[All Fields] OR "depressively"[All Fields] OR "depressiveness"[All Fields] OR "depressives"[All Fields]<br>Neoplasms: "neoplasm's"[All Fields] OR "neoplasms"[MeSH Terms] OR "neoplasms"[All Fields] OR "neoplasm"[All Fields]<br>Patients: "patient's"[All Fields] OR "patients"[MeSH Terms] OR "patients"[All Fields] OR "patient"[All Fields] OR "patients's"[All Fields]<br>Gender Role: "gender role"[MeSH Terms] OR ("gender"[All Fields] AND "role"[All Fields]) OR "gender role"[All Fields]	109
**Scopus**<br>"Depression" AND "Neoplasms" AND "Patients" AND "Gender Role" AND (LIMIT-TO (DOCTYPE , "ar")) AND (LIMIT-TO (LANGUAGE , "English"))	121
**WOS**<br>Depression (All Fields) and Neoplasms (All Fields) and Patients (All Fields) and Gender role (All Fields)	11
